# A Single Case Report of Granular Cell Tumor of the Tongue Successfully Treated through 445 nm Diode Laser

**DOI:** 10.3390/healthcare8030267

**Published:** 2020-08-13

**Authors:** Maria Vittoria Viani, Luigi Corcione, Chiara Di Blasio, Ronell Bologna-Molina, Paolo Vescovi, Marco Meleti

**Affiliations:** 1 Department of Medicine and Surgery, University of Parma, 43126 Parma, Italy; lcorcione@ao.pr.it (L.C.); paolo.vescovi@unipr.it (P.V.); marco.meleti@unipr.it (M.M.); 2 Private practice, Centro Medico Di Blasio, 43121 Parma; Italy; chiaradb@hotmail.it; 3 Faculty of Dentistry, University of the Republic, 14600 Montevideo, Uruguay; ronellbologna@hotmail.com

**Keywords:** granular cell tumor, diode laser, pseudoepitheliomatous hyperplasia, oral surgery

## Abstract

Oral granular cell tumor (GCT) is a relatively rare, benign lesion that can easily be misdiagnosed. Particularly, the presence of pseudoepitheliomatous hyperplasia might, in some cases, lead to the hypothesis of squamous cell carcinoma. Surgical excision is the treatment of choice. Recurrence has been reported in up to 15% of cases treated with conventional surgery. Here, we reported a case of GCT of the tongue in a young female patient, which was successfully treated through 445 nm diode laser excision. Laser surgery might reduce bleeding and postoperative pain and may be associated with more rapid healing. Particularly, the vaporization effect on remnant tissues could eliminate GCT cells on the surgical bed, thus hypothetically leading to a lower rate of recurrence. In the present case, complete healing occurred in 1 week, and no recurrence was observed after 6 months. Laser surgery also allows the possibility to obtain second intention healing. Possible laser-induced histopathological artifacts should be carefully considered.

## 1. Introduction

Granular cell tumor (GCT), also called the Abrikosoff tumor, is a relatively rare benign lesion, presumptively originating from Schwann cells. GCT has been described for the first time by Abrikosoff in 1926 and named “myoblastic tumor”, based on its supposed origin from muscle cells [1].

The estimated incidence of oral GCT is approximately 1:1,000,000 population per year [2]. The tumor is relatively more common among black people [3]. GCT can affect people at any age, but it is commonly diagnosed between the second and sixth decades of life [4]. Some studies show a predilection for females [5].

GCT can be found in any part of the body. Common locations are skin, respiratory tract, breast, gastrointestinal tract. Up to 50% of lesions have been reported to arise in the head and neck region, with a peculiar predilection for the tongue. Other oral sites of occurrence are the lips, the retrocomissural area, and buccal mucosa, followed by the palate, uvula, and parotid gland [4].

Oral GCT typically appears as a single, sessile, asymptomatic whitish nodule that gradually increases in size. Cases of multiple lesions have been reported [5]. Tumor surface is usually smooth, with few cases presenting ulceration. Size is commonly less than 3 cm. Pain is uncommon, but some patients may experience discomfort during tooth brushing, eating spicy food, and oral trauma [6].

Malignant transformation has rarely been reported, and only 2% of cases have documented to metastasize to distant sites [4].

Very few cases of GCT, successfully managed through different types of laser, have been reported in the literature, but no one of these using diode laser.

The first application of diode laser in dentistry dates back in the mid-90s. Common ranges of wavelengths cover 810, 940 and 980 nm. Many advantages have been described in association with the use of diode laser: definite cutting edge, hemostasis, no need for suturing, reduction in surgical time, less post-surgical pain, and ease of use. Disadvantages reported in the literature include delayed wound healing and possible carbonized borders of the surgical field [7].

Here, we reported a case of GCT of the tongue in a young female patient, which was successfully treated through 445 nm diode laser excision.

## 2. Materials and Methods

A 35-year-old white woman was referred by her dentist to the section of Oral Medicine and Oral Surgery of the University of Parma, Italy, because of the presence of a painless, solitary nodule on the dorsum of the tongue.

The patient reported that the swelling was initially small, and it gradually increased in size over the last few months.

**Figure 1:**
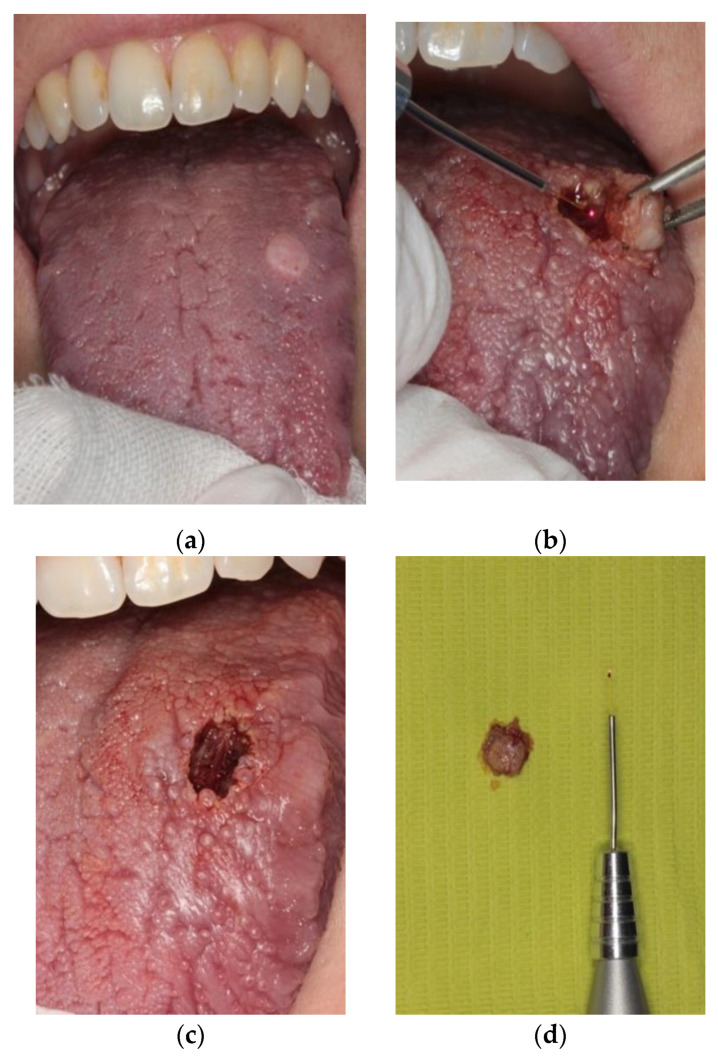
(**a**) Clinical appearance of granular cell tumor of the left dorsum of the tongue. (**b**) Surgical excision of the lesion with 445 nm diode laser. (**c**) Immediate post-surgical appearance. Complete hemostasis. (**d**) Surgical specimen.

At the clinical evaluation, it was noticeable a well-defined, non-tender, and non-fluctuant whitish lesion, with smooth borders, on the left side of the dorsum of the tongue. The lesion measured approximately 1 cm. The days before, the patient reported small ulceration, which could not be seen on the day of surgery (Figure 1).

Differential diagnosis included pyogenic granuloma, GCT, lipoma, and traumatic fibroma of unknown cause.

The patient reported no history of smoking and alcohol consumption, and the anamnesis was negative for systemic disorders.

The lesion was excised through a diode laser (K Laser Blue Dental^©^, Eltech K-Laser s.r.l., Treviso, Italy, 3.5 W, continuous wave, 445 nm) under local anesthesia, including 0.2 cm of surrounding clinically healthy tissue (Figure 1b–d).

Taking into account the likely benign nature of the lesion, we chose to perform an excision with about 1 mm of clinically healthy tissue around the nodule, including deep tissues.

Informed consent was obtained by the patient (provided as Supplementary Material).

## 3. Results

Histopathological examination revealed, in the connective compartment, the presence of large, spindle, oval to polygonal cells with small, uniform eosinophilic granules and small round to oval nuclei, which stained strongly positive to S-100 protein. Nuclei were small, all prominent, vesicular, and dark. Cells, arranged in nests, sheets, and fascicles, were not encapsulated; they permeated the collagen fibers, skeletal muscle, adipocytes, blood vessels, and nerves. The overlying epithelium was slightly hyperplastic with focal features of pseudoepitheliomatous hyperplasia that could provide confusion with squamous cell carcinoma; however, lack of nuclear hyperplasia or pleomorphism and the presence of granular cells could differentiate this condition.

Vascularity was prominent in most cases.

No cellular atypia or mitotic figures were seen.

**Figure 2:**
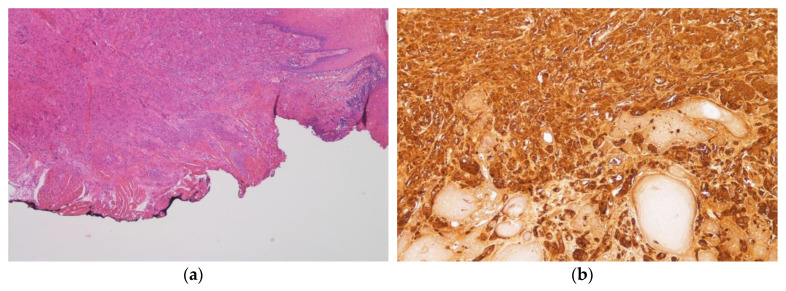
(**a**) Histopathological features of granular cell tumor. The massive presence of granular cells (H&E staining; 10× magnification). (**b**) Immunohistochemical staining for S-100 protein. Strong positivity (40× magnification).

We also noticed one important aspect that differentiated a GCT from a malignant tumor: chronic inflammatory cells were intensively present in squamous cell carcinomas, but not seen in GCT (Figure 2). On the basis of such histopathological features, a final diagnosis of GCT was rendered.

Ferreira et al. also underlined the importance of the immunohistochemical panel in determining the biological aspects of the lesion and the final diagnosis. The specific antibodies used were S-100, vimentin, CD68, p53, Ki-67, E-cadherin, collagen IV, and cytokeratin AE1/AE3. Their results showed a strong nuclear and cytoplasmic staining of S-100 protein and vimentin. CD68 revealed strong staining in the cytoplasm of granular cells. The expression of p53 was found in the nuclei of basal and parabasal cells of normal epithelium, as well as diffuse staining in the granular cells. In addition, cells of the basal layer of the epithelium were weakly marked with Ki-67. E-cadherin showed a uniform “membranous staining” in the overlying epithelium. Some regions of the endothelium were marked with collagen IV. Finally, granular cells were negative for cytokeratin AE1/AE3 [5].

**Figure 3:**
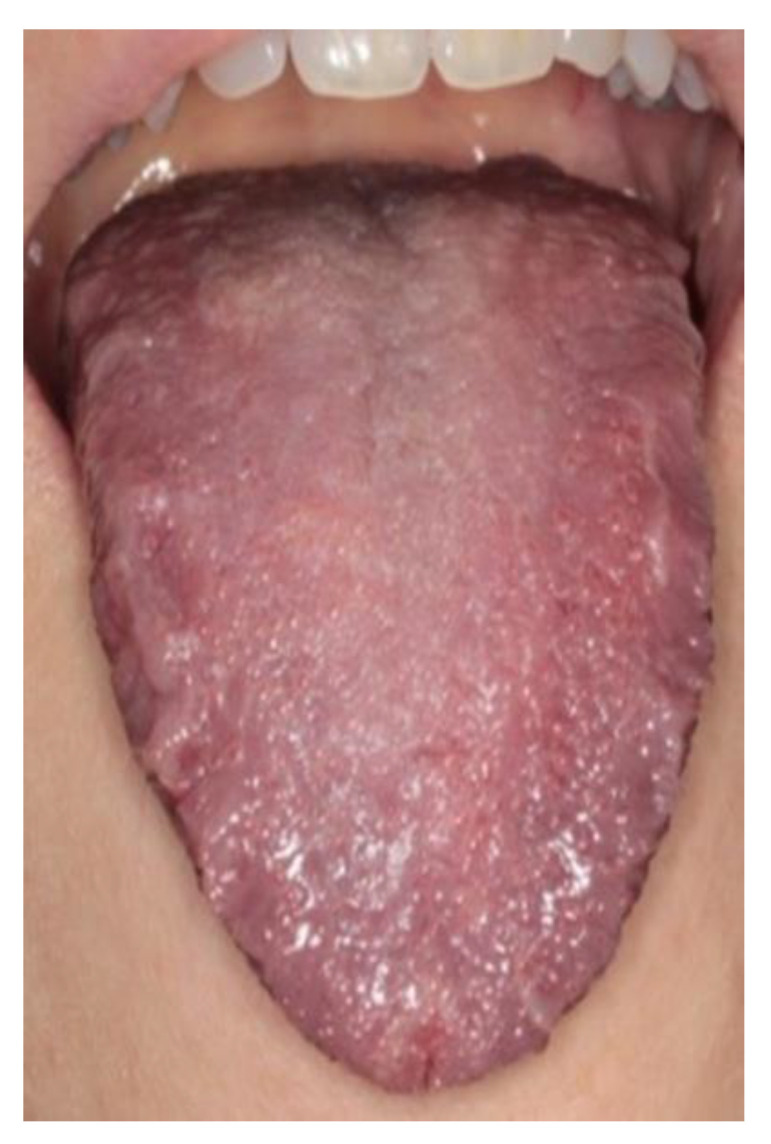
One-week follow-up. Complete healing.

Complete healing occurred in 1 week, and no recurrence was observed in the admittedly short follow-up of 6 months (Figure 3).

## 4. Discussion

Granular cell tumor is a rare, benign tumor that can be easily misdiagnosed, particularly by clinicians with no experience in oral medicine and/or oral surgery.

Differential diagnoses reported in the literature are somewhat heterogeneous and include relatively common lesions, such as squamous cell carcinomas, soft-tissue cysts, pyogenic granuloma, fibroma, traumatic lesions, as well as more peculiar diseases, such as schwannoma, giant sialolith, pleomorphic adenoma of minor salivary glands, congenital epulis of newborn, leiomyoma, rhabdomyoma, oncocytoma, melanocytic nevi, and metastatic lesions [8,9,10].

From the histological point of view, GCT is non-encapsulated, and it is typically composed of polygonal eosinophilic cells with central, small, dark nuclei and abundant granular cytoplasm. Such cells permeate the collagen fibers, and they stain positive for S-100 and vimentin, which can be used to confirm the pathology [4]. A very peculiar and fascinating histological phenomenon that can be seen in GCT (and in other oral lesions) is the so-called pseudoepitheliomatous hyperplasia, a proliferation of the epithelium with irregular nests and chords of squamous cells that can be dispersed in the surrounding connective tissue, with a pattern vaguely resembling the architecture of a malignant epithelial tumor [11]. However, the epithelial cells of pseudoepitheliomatous hyperplasia, typically, do not display signs of atypia [4]. Moreover, the connective tissue around GCT does not show the presence of inflammation, as it happens for many malignant lesions.

Many examples of laser treatment of GCT are reported in the literature.

Sproat et al. detailed a case of GCT of the larynx excised with a CO_2_ laser. Follow-up was characterized by complete recovery and absence of recurrence [12]. Crippa et al. presented 11 cases of GCTs treated with 808 and 830 nm laser with no recurrence during 5 years follow up [13].

Romeo et al. described the surgical treatment of two cases on the dorsum and margins of the tongue; KTP laser (SmartLite, DEKA, Calenzano, Italy, 1.5 W, 100 ms, fluence 212 J/cm^2^) was used in both cases; such device induced effective hemostasis, which allowed to avoid sutures. According to the experience of these authors, post-surgical pain was lower than that observed after intervention performed through the cold blade. Besides, the surgical excision was faster and easier than the traditional one [14].

Schneider et al. reported a procedure of endoscopic Nd:YAG laser (Fotona, Lublijana, Slovenia) photocoagulation for the treatment of GCT of the esophagus; these authors also highlighted an apparent lower post-surgical discomfort [15].

The other four cases of bronchial GCT were conservatively treated through Nd:YAG laser by van der Maten et al. All patients underwent complete recovery, and they did not develop recurrences during follow-up [16]. Piazza et al. presented two cases of GCT of the hypopharynx treated by endoscopic CO_2_ laser excision, highlighting the possibility to work in a bloodless surgical field and to ensure a precise and safe resection of the tumor [17].

With regard to lasers that have a possible application in oral soft tissue surgery, Er:YAG laser (Fotona, Lublijana, Slovenia, wavelength: 2940 nm) penetrates up to 0.1 mm and has no effect of carbonization. Such laser is absorbed by hydroxyapatite and water, and it is, therefore, primarily indicated for hard tissue surgery. Nevertheless, several authors have used Er:YAG laser for cutting and ablating soft tissue with satisfying results. The surgery performed with such laser has proved to be efficient and provides clean and high precision cut with minimal injury to the adjacent hard and soft tissue. It has also the advantage of inducing a lower increase in temperature of surrounding tissues when compared to traditional burs [11]. The hemostatic effect of Er:YAG laser is, however, usually limited as its use is based on water and air cooling [11].

KTP laser (wavelength: 532 nm) can produce stromal changes and vascular damages if not used correctly. The affinity for hemoglobin allows, in most cases, to use lower power and fluence [14].

CO_2_ laser (wavelength: 10.600 nm) has, to some extent, a low versatility and can lead to an excessive scaring retraction and a higher post-surgical discomfort.

To the best of our knowledge, this is the first case report of a GCT of the tongue treated with 445 nm diode laser.

The choice of 445 nm diode laser, also called “blue laser”, was based upon recent evidence in literature; a study of Reichelt et al. evaluated the effects of such laser on a cellular level. They described a large wound due to the high temperature, but fast healing. This wavelength seems not associated with particular side effects on cells, tissues, cytoskeleton, and DNA [18].

The 445 nm diode laser demonstrates photoangiolytic properties comparable with KTP laser, power of carbonization, and coagulation. It combines cutting properties of diode laser with the coagulation vessel capability of KTP. Other advantages listed by Hess et al. are the portability of the shoe-box, the versatility of such laser in many surgery fields, the possibility to work with continuous-wave for less than a millisecond, a better cutting than KTP [19].

Treatment of GCT consists of surgical excision, possibly with disease-free margins. Recurrence can occur many years after primary tumor resection, and it is most probably related to not radical excision. Besides, late occurrence in other parts of the body has been documented (metachronous lesions) [2].

Benign granular cell tumors have a recurrence rate of 2 to 8% when resection margins are deemed clear of tumor infiltration and are increased to 20% when the resection margins of a benign granular cell tumor are positive for tumor infiltration [20].

We hypothesize that the use of diode laser for surgical excision of GCT may reduce the risk of recurrence. In fact, such laser has the ability to penetrate within the oral soft tissues from 0.27 mm up to 3.94 cm, and such effect might contribute to ablate possible cellular remnants of the tumor, which are believed to be the potential cause of recurrence [21]. However, our hypothesis is based only on the present case report, and, therefore, it does not have a scientific value.

A reduction in the risk of recurrence was confirmed also by Rai et al. in the treatment of pyogenic granuloma (recurrence rate: 16%). The authors used a diode laser (KaVo Dental GmbH, Washington, DC, USA) with 808 nm wavelength, 7 W power, in continuous/interrupted pulse mode. No recurrence was observed after such treatment [22].

Andreadis et al. also highlighted no recurrences for pyogenic granuloma radically treated with diode laser [23].

The risk of recurrence has also been reduced after laser treatment of oral leukoplakia, which, in general, is regarded as a lesion with a high tendency to recur [24].

However, it is very important to carefully consider the extension of the incision within the surrounding tissues, in order to avoid laser-induced artifacts on tumor margins, which are possibly associated with histopathological misdiagnosis. A disadvantage of a diode laser, indeed, is the increase in temperature, described also by Fornaini et al.; lower temperatures are described for Er:YAG and KTP laser in contrast to Nd:YAG and diode laser [25,26].

This may lead to problems in excision margins. Common artifacts that may influence the histopathological diagnosis include epithelial modifications (e.g., nuclear and cytoplasmic distortion, epithelial detachment, and connective alterations (e.g., thermal damages and elastosis) [26]. On the other hand, a study conducted by Palaia et al. demonstrated that the laser damage was lower than 1 mm, and the diode laser did not compromise the histopathological analysis of benign lesions. Nevertheless, in suspicious lesions, a margin of 1 mm was recommended [27].

Monteiro et al. demonstrated that the highest tissue damage was found on the tissue excised with the electrosurgical scalpel, followed by a diode laser, Nd:YAG laser, CO_2_ laser, Er:YAG laser, and the scalpel; nevertheless, the histopathological readability of specimens was not compromised in any case [28].

Because of its wavelengths, the diode laser is poorly absorbed in hard tissues, and it could not be used for bone and dental lesions [29].

An important advantage of diode laser surgery on oral soft tissue is the possibility to achieve effective hemostasis, which allows avoiding sutures in most cases, thus leading to second intention healing [30].

Pain and post-surgical discomfort are usually lower for interventions performed with lasers than for those with a cold blade. The biophysical mechanisms underlying such differences are believed to depend on the antimicrobial and photobiomodulative effects of laser beams [30].

The diode laser is a very versatile device; as Al-Mohaya et al. stated, such laser can be used for surgery in uncontrolled diabetic patients, in which a bloodless surgical site and excellent post-operative healing are important [31].

The diode laser can also be very useful in patients with hemostasis disorders or in therapy with oral anticoagulants, in order to enhance coagulation, or in patients with hypertension [32].

Other methods of use of diode laser include the photocoagulation of vascular lesions and bleaching of pigmentations. Such effects are based on the high affinity of laser wavelength for melanin and hemoglobin.

Other possible uses include the so-called photobiomodulation, also known as low-level laser therapy (LLLT).

For example, in a study by Akbulut et al., diode laser LLLT was used for oral lichen planus lesions, not responding to conventional steroid treatment, and for mucous membrane pemphigoid [29].

Monteiro et al. also reported that diode laser reduced recurrence and malignant transformation of oral leukoplakia [33].

Diode laser finds application also in the case of physiologic gingival pigmentation; the high affinity for hemoglobin and melanin makes such laser the best device for depigmentation of gingiva [34].

## 5. Conclusions

In conclusion, the present case gives support to the hypothesis that 445 nm laser is a reliable tool for the surgical excision of GCT and similar benign lesions of the oral mucosa. Such a device is probably associated with the possibility of reducing the risk of recurrences. Moreover, laser surgery has a well-established utility in reducing post-surgical pain and increasing the clinician comfort, eliminating, in most cases, the intraoperative bleeding [30]. It is important to highlight here that more long-term prospective studies are needed both at clinical and histopathological levels in order to establish the advantages of laser surgery on oral soft tissues. Particularly, the reliability of laser treatment of potentially malignant disorders and malignant lesions should be confirmed by long-term follow-up clinical randomized trials.

## Supplementary Materials

The following are available online at https://www.mdpi.com/2227-9032/8/3/267/s1, Supplementary material: Informed consent.
