# Supplementary figures and images for: A Single Case Report of Granular Cell Tumor of the Tongue Successfully Treated through 445 nm Diode Laser
Source: Healthcare (Basel). 2020 Aug 13;8(3):267. doi: 10.3390/healthcare8030267 (PMC7551446; doi:10.3390/healthcare8030267)

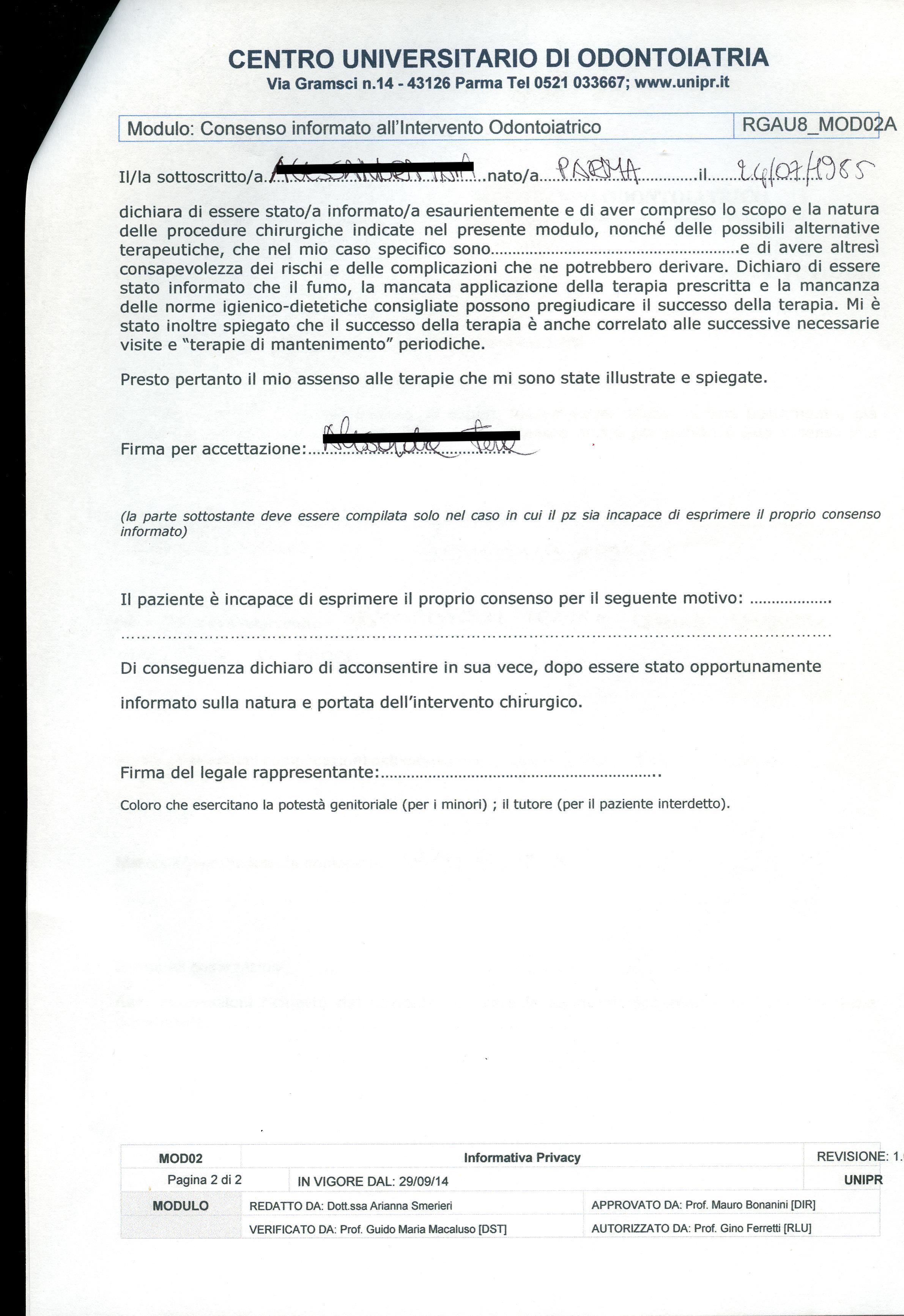

Supplement: Supplementary file 1 [file healthcare-08-00267-s001.zip › informed consent.jpg]

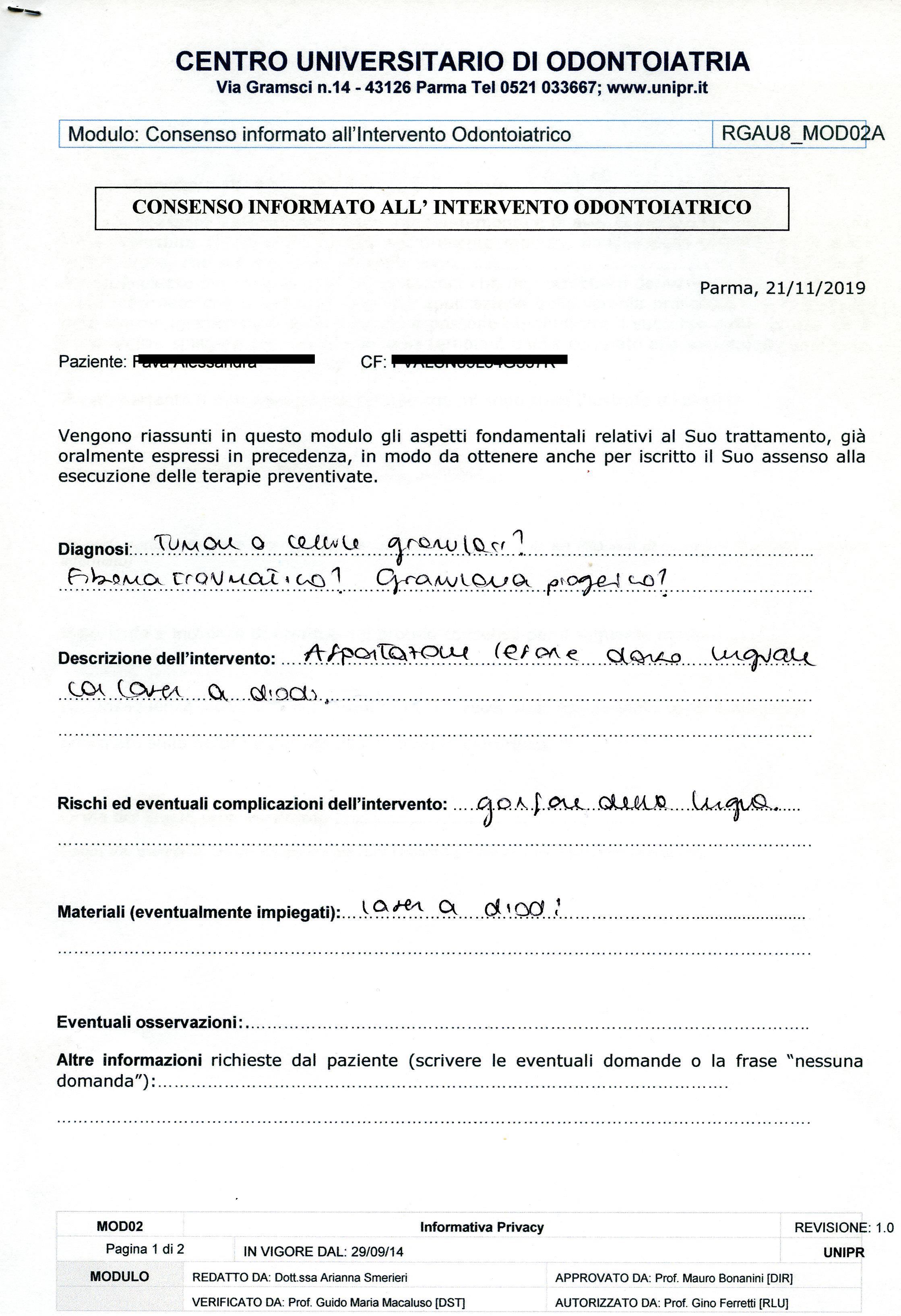

Supplement: Supplementary file 1 [file healthcare-08-00267-s001.zip › informed consent 2.jpg]
